# Cooperative but distinct early co-signaling events originate from ERBB2 and ERBB1 receptors upon trastuzumab treatment in breast cancer cells

**DOI:** 10.18632/oncotarget.17686

**Published:** 2017-05-08

**Authors:** Paola Bagnato, Alessia Castagnino, Katia Cortese, Maria Bono, Silvia Grasso, Grazia Bellese, Tiziana Daniele, Richard Lundmark, Paola Defilippi, Patrizio Castagnola, Carlo Tacchetti

**Affiliations:** ^1^ DIMES, Dipartimento di Medicina Sperimentale, Anatomia Umana, Università di Genova, Genova, Italy; ^2^ Molecular Biotechnology Centre and Department of Genetics, Biology and Biochemistry, Torino, Italy; ^3^ San Raffaele Scientific Institute, Experimental Imaging Centre, Milan, Italy; ^4^ Department of Medical Biochemistry and Biophysics, Umea University, Umea, Sweden; ^5^ Department of Integrated Oncological Therapies, IRCCS AOU - San Martino - IST, Largo Rosanna Benzi, Genova, Italy

**Keywords:** ERBB2, trastuzumab, circular dorsal ruffles, ERBB1, breast cancer

## Abstract

ERBB2 receptor belongs to the ERBB tyrosine kinase receptor family. At variance to the other family members, ERBB2 is a constitutively active orphan receptor. Upon ligand binding and activation, ERBB receptors form homo- or hetero-dimers with the other family members, including ERBB2, promoting an intracellular signaling cascade. ERBB2 is the preferred dimerization partner and ERBB2 heterodimers signaling is stronger and longer acting compared to heterodimers between other ERBB members. The specific contribution of ERBB2 in heterodimer signaling is still undefined.

Here we report the formation of circular dorsal ruffles (CDRs) upon treatment of the ERBB2-overexpressing breast cancer cell lines SK-BR-3 and ZR751 with Trastuzumab, a therapeutic humanized monoclonal antibody directed against ERBB2. We found that in SK-BR-3 cells Trastuzumab leads to surface redistribution of ERBB2 and ERBB1 in CDRs, and that the ERBB2-dependent ERK1/2 phosphorylation and ERBB1 expression are both required for CDR formation. In particular, in these cells CDR formation requires activation of both the protein regulator of actin polymerization N-WASP, mediated by ERK1/2, and of the actin depolymerizing protein cofilin, mediated by ERBB1. Furthermore, we suggest that this latter event may be inhibited by the negative cell motility regulator p140Cap, as we found that p140Cap overexpression led to cofilin deactivation and inhibition of CDR formation.

In conclusion, here we show for the first time an ERBB2-specific signaling contribution to an ERBB2/ERBB1 heterodimer, in the activation of a complex biological process such as the formation of CDRs.

## INTRODUCTION

ERBB2 (Her2/Neu) is a member of the ERBB family of receptor tyrosine kinases (RTKs), which includes EGFR (ERBB1), ERBB3, and ERBB4. ERBB2 is an orphan receptor and is the preferred dimerization partner for ERBB1, 3, and 4, upon their activation following the binding to specific ligands, e.g. EGF, TGF-α, and amphiregulin (for EGFR), or Heregulins/Neuregulins (for ERBB3/4) [1–3]. The oncogenic signaling by ERBB2 [4, 5] is thought to involve the sustained activation of a number of signaling pathways, including the Ras-Raf-MAPK, which contributes to cell proliferation, and the PI3K-AKT, which induces cell survival [2, 6, 7].

Relative ratio of ERBB2/ERBB1 heterodimers on the plasma membrane varies among breast cancer cell lines; in the widely used SK-BR-3 line it was found to be approximately 3.2 [8]. However, the precise contribution of ERBB2 to the heterodimer signaling is not clear yet. In particular, evidence suggests that in the absence of ERBB2 containing heterodimers, ERBB agonists elicited a weaker and curtailed signaling response [9].

The binding of the extracellular juxta-membrane region of ERBB2 to the therapeutic humanized monoclonal antibody Trastuzumab (Tz) [10, 11] in ERBB2 overexpressing cells, results in the down regulation of the PI3K-AKT signaling, ERK1/2 activation in SK-BR-3 cells [12], increased nuclear accumulation of the cell cycle inhibitor p27^Kip1^, and cell cycle arrest [13, 14]. At variance from earlier reports [15, 16], it is now opinion that endocytic down regulation of ERBB2 does not represent a relevant mechanism for signaling attenuation mediated by Tz [17, 18].

Circular dorsal ruffles (CDRs), also known as dorsal waves, are plasma membrane protrusions that merge in a single ring-shaped projection erecting vertically from the dorsal cell surface [19, 20]. These protrusions are formed as a result of the assembly of highly dynamic cortical actin-based structures in response to different growth factors [19, 21]. In contrast to other plasma membrane (PM) protrusions, CDRs are transient, and disappear usually within 5-30 min from the application of appropriate stimuli [22–24], such as ligands for several RTKs, ERBB1 included [19]. The formation of CDRs is, therefore, initiated by RTKs signaling and involves a complex array of reactions, since actin polymerization is induced by the Arp 2/3 activation by the Wiskott-Aldrich syndrome (WAS) family of proteins N-WASP and WAVE. Furthermore, activation of N-WASP is enhanced by ERK1/2-dependent phosphorylation of cortactin [25, 26], an actin-binding protein involved in polymerizarion of actin filaments necessary for the remodeling of actin cortical structures in CDR formation [19]. Moreover, cofilin, an actin-binding protein which disassembles actin filaments [27] and drives PM extension [28] might be implicated in CDR disassembly.

Although the functions of CDRs have not been conclusively clarified, it has been suggested that CDRs induce internalization of RTKs after ligand stimulation, macropinocytosis, and a rapid reorganization of the actin cytoskeleton preparing for cell motility [19, 29]. Despite cancer cells form less CDRs as compared to non transformed cells [19], CDR-related mesenchymal-like migration in 3-dimensional matrices has been associated with cancer metastasis [19], but their possible clinical relevance remains unclear.

In this study, we show that Tz treatment of the ERBB2-overexpressing breast cancer cells, promotes ERBB2/ERBB1 heterodimers and CDR formation. Taking advantage of this biological response, we identified and characterized two distinct and spatiotemporally coordinated ERBB2 and ERBB1 signaling pathways leading to CDR formation, thus dissecting the specific activity of ERBB2 in an ERBB2/ERBB1 heterodimer, in the absence of specific ERBB1 signaling.

## RESULTS

### Tz induces dramatic remodeling of the plasma membrane and circular dorsal ruffles formation

**Figure 1:**
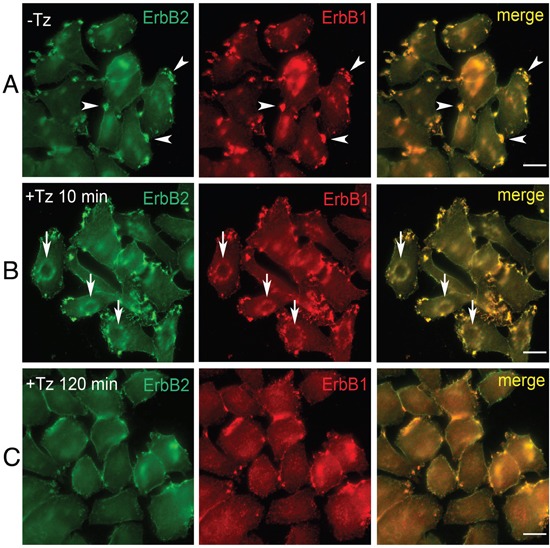
Trastuzumab (Tz) treatment modifies ERBB2 plasma membrane (PM) distribution and induces the formation of Circular Dorsal Ruffles (CDRs) SK-BR-3 control cells (NT) and SK-BR-3 cells treated with Tz at various time points, were fixed and immunostained for ERBB2 (green signal) and ERBB1 (red signal). Three different distribution patterns of PM ERBB2/ERBB1 were recognized **(A, B, C)**. Arrowheads indicate peripheral patches **(A)**; arrows indicate CDRs **(B)**, while **(C)** shows diffuse membrane localization. Scale bars = 10 μm. **(B)** Cortical dorsal ruffles (CDRs) were observed after 10 and 15 min of Tz treatment.

To characterize early events occurring at the level of the PM upon Tz binding to ERBB2, we analyzed the breast cancer cell line SK-BR-3, in a time range between 2 and 120 min of treatment with Tz. ERBB1 and Tz-bound ERBB2 distribution was revealed by immunofluorescence on fixed cells, using antibodies directed to human IgGs and ERBB1, respectively (Figure 1). While in untreated SK-BR-3 cells, ERBB2 and ERBB1 were mainly organized in clusters/patches at the cell periphery (Figure 1A), after 10 min of Tz the two receptors co-redistributed in *bona fide* CDRs (Figure 1B), and displayed a diffuse distribution on the PM after 120 min of treatment (Figure 1C). As expected, a diffuse co-redistribution of ERBB2 and ERBB1 on the PM was also observed upon EGF treatment after 20 min when the percentage of cells showing CDRs is negligible or absent (Supplementary Figure 1).

**Figure 2:**
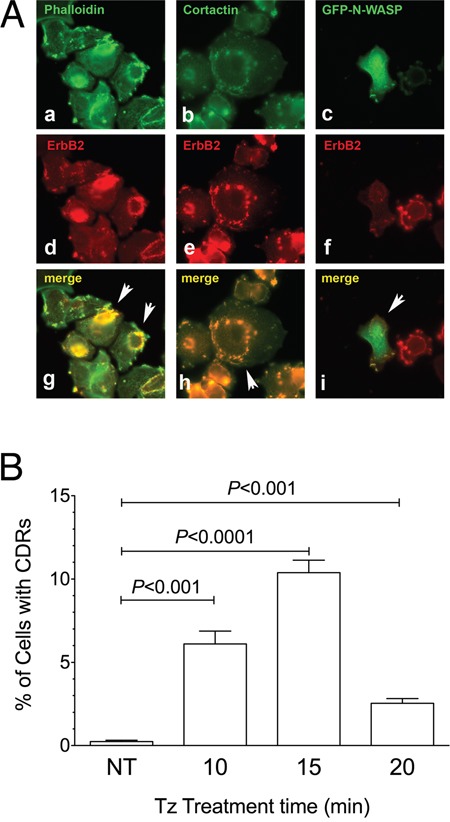
Cortactin and N-WASP are recruited on Circular Dorsal Ruffles (CDRs) that are transient PM structures induced by Trastuzumab (Tz) **(A)** SK-BR-3 were treated with Tz for 15 minutes, fixed and processed for immunofluorescence. **(a)** F-Actin stained with fluorescent phalloidin; **(b)** Cortactin revealed with a specific antibody; **(c)** N-WASP:GFP chimera revealed in the green channel; **(d, e, f)** ERBB2 detected with anti-ERBB2 antibody. Arrowheads indicate colocalization of F-actin, cortactin and N-WASP:GFP green signals with ERBB2 red signals in CDRs **(g, h, i)**. Scale bars = 10 μm. **(B)** Quantification of Tz-induced CDR formation in SK-BR-3 control cells (NT) and SK-BR-3 cells treated with Tz at various time points indicated in min. Bars represent the average ± SD of the data pooled from 3 independent experiments. At each time point at least 500 cells were analyzed.

The co-localization in SK-BR-3 cells of ERBB2 with F-actin and with CDR markers such as cortactin and N-WASP:GFP chimera [34] confirmed that the observed structures were indeed CDRs (Figure 2A) as well as the colocalization of cortactin with F-actin and ERBB2 (Supplementary Figure 2). The percentage of cells displaying CDRs on their PM was significantly higher in the 10-15 min range of Tz treatment, compared to untreated cells (P<0.001 and P<0.0001, respectively) (Figure 2B). In our experimental setting, after 20 min of Tz treatment the percentage of cells showing CDRs was much reduced or negligible (Figure 2B). Similar results were obtained with an additional human breast cancer cell line named ZR751, which also overexpresses ERBB2, and showed colocalization of cortactin with F-actin and ERBB2 and a peak of CDR formation at 15 min followed by a decrease at 20 min of Tz treatment (Supplementary Figure 3).

### Tz-induced CDRs depend upon ERK1/2 activation

**Figure 3:**
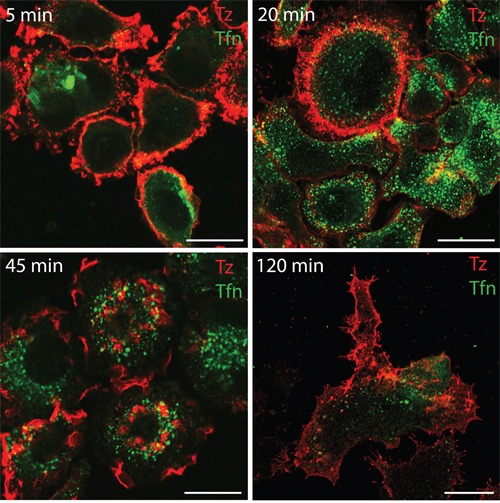
Transferrin but not Tz induces receptor internalization within 120 min of treatment in SK-BR-3 cells SK-BR-3 cells were treated with Alexa555-conjugated Tz (red signal) and with Alexa488-conjugated transferrin (green signal), fixed and imaged by confocal microscopy at the indicate time points (upper left corner).

To investigate whether in our system CDRs represent a PM domain responsible for ERBB2 endocytosis or a signaling platform, we first compared the internalization of this receptor with that of the transferrin receptor after transferrin binding by immunofluorescence analysis. Our results showed that ERBB2 internalization upon Alexa555-Tz administration is negligible compared to that occurring to the Alexa488-transferrin (Figure 3). Therefore, we hypothesized that ERBB2 redistribution at the cell surface and CDR induction may be more related to signaling events rather than endocytosis.

**Figure 4:**
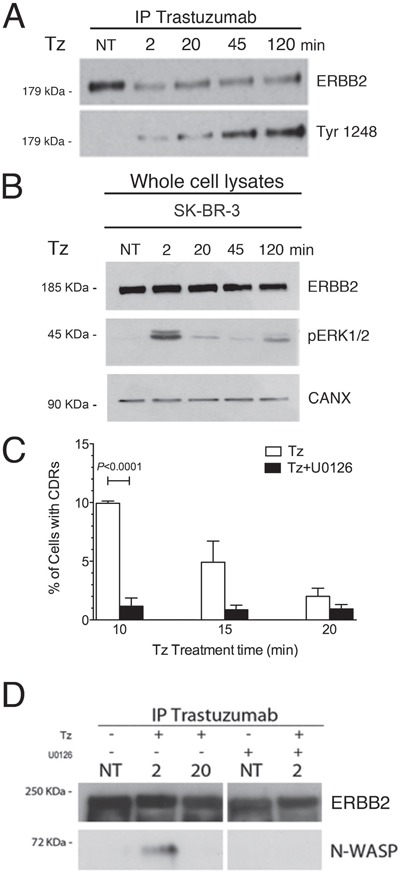
Trastuzumab (Tz) induces ERBB2 phosphorylation and activation of ERK 1/2 signaling which is necessary for N-WASP binding and CDRs formation **(A)** Control SK-BR-3 (NT) or Tz treated at different time points as indicated (in min) were harvested and Tz bound ERBB2 was immunoprecipitated and blotted to analyze the extent of Tyr 1248 phosphorylation. **(B)** ERK1/2 phosphorylation (pERK1/2) levels were analyzed in total cell lysates and calnexin (CANX) was used as loading control. **(C)** SK-BR-3 were treated with Tz (white bars) or with Tz along with the MEK inhibitor U0126 (black bars) and cells displaying CDRs were scored at each indicated time point. Bars represent the average ± SD of the data pooled from 3 independent experiments. At each time point at least 500 cells were analyzed. **(D)** ERBB2 bound to Tz was immunoprecipitated and then endogenous N-WASP and ERBB2 were detected by western blot analysis in cells treated with Tz and/or with U0126 at various time points indicated in min.

Indeed, CDR formation is a complex event involving a signaling-mediated actin remodeling. Tz is known to promote the phosphorylation of the ERBB2 kinase domain, and ERK1/2 activation in SK-BR-3 cells [12]. To assess the timing of these events in our experimental context, control untreated and Tz-treated SK-BR-3 cell lysates were processed for Tz IP and immunoblot analysis. We observed that ERBB2 Tyr1248 phosphorylation increased progressively from 2 to 120 min (Figure 4A), whereas ERK1/2 phosphorylation showed a peak after 2 min of Tz treatment (Figure 4B).

Since the ERK1/2 pathway is involved in actin nucleation, cytoskeleton reorganization [35], and activation of the cortactin-Arp2/3-N-WASP complex, involved in CDR formation [26], we tested whether the inhibition of the ERK1/2 activation would impair CDR formation induced by Tz. To this aim, we challenged the SK-BR-3 cells with the MEK1 specific inhibitor U0126, in combination with Tz, and scored the percentage of cells displaying CDRs. Under these conditions we detected a significantly lower fraction of cells displaying CDRs, after 10 min of treatment, compared to cells cultured in the absence of U0126 (Figure 4C). To assess whether ERBB2 interaction with N-WASP occurs after Tz treatment and depends from ERK1/2 activity, we performed co-IP studies in the absence and presence of U0126. The results revealed that the endogenous N-WASP co-immunoprecipitated with ERBB2 at 2 min (Figure 4D), whereas this association was lost after 20 min, suggesting that the molecular machinery for CDR formation was transiently assembled at earlier time points of Tz treatment and mostly disassembled at 20 min. Furthermore, we showed that the addition of U0126 impaired N-WASP co-IP with ERBB2 (Figure 4D). Overall, these results show that Tz-induced ERK1/2 signaling is necessary to promote CDR formation.

### ERBB1 is necessary for CDR formation induced by Tz

As immunofluorescence data showed a complete co-distribution of ERBB2 with ERBB1 in the CDRs, upon Tz treatment (Figure 1A), we decided to evaluate the specific contribution of the signaling downstream of either ERBB2 or ERBB1 in the CDR formation process.

**Figure 5:**
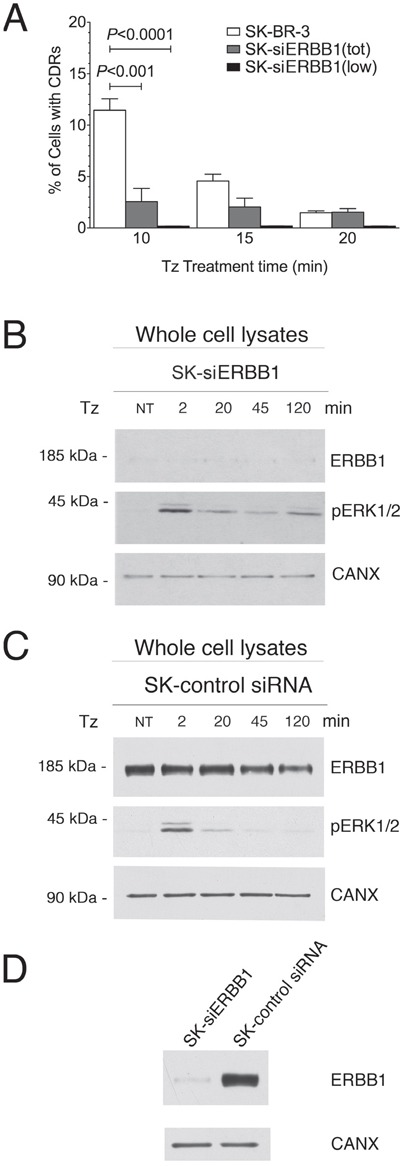
ERBB1 is crucial for the CDR formation. SK-BR-3 wt or SK-BR-3 cells transfected with the siRNA #1 (see Material and Methods) specific for ERBB1 (SK-siERBB1) were treated with Tz for various times as indicated (in min) **(A)** Histogram of the percentage of cells showing CDRs at each time point. Bars represent the average ± SD of the percentage of cells with CDRs, observed at each time point pooled from 3 independent experiments. At each time point at least 500 cells were analyzed. The analysis was performed in either the total population of SK-BR-3 and SK-siERBB1 (tot) or only on those SK-siERBB1 cells showing very low or no ERBB1 signal (low). **(B)** Immunoblot analysis of ErbB1 expression and ERK1/2 phosphorylation (pERK1/2) in SK-siERBB1 cells. **(C)** Immunoblot analysis of ERBB1 expression and ERK1/2 signaling in SK-BR-3 cells transfected with a control siRNA (SK-control siRNA). **(D)** Immunoblot analysis of ERBB1 expression in SK-siERBB1 and SK-control siRNA. Calnexin (CANX) was used as loading control.

To this end, we silenced ERBB1 in SK-BR-3 by RNA interference, using the ERBB1 siRNA #1 (SK-siERBB1) by RNA interference and observed a dramatic reduction of the percentage of cells displaying CDRs in the overall population of SK-siERBB1 compared to control SK-BR-3 cells (P<0.001) (Figure 5A). The impairment of CDR formation was even more evident when only those cells showing low expression or no expression at all of ERBB1 in the SK-siERBB1 cell population were taken into account (P<0.0001) (Figure 5A). Experiments performed using a different ERBB1 siRNA (ERBB1 siRNA #2) yielded similar results (Supplementary Figure 4) and we ruled out by immunofluorescence analysis that ERBB2 on the plasma membrane was lost in SK-siERBB1 cells (Supplementary Figure 5). These results suggested that ERBB1 promotes CDR induction by Tz.

As we observed a strong dependence of CDR formation on ERK1/2 signaling induced by Tz, we investigated whether the activation of this pathway depended on ERBB1. We found that SK-siERBB1 cells and those transfected with a control scrambled siRNA, displayed the same pattern of ERK1/2 activation upon Tz treatment (Figure 5B, 5C), which was also similar to that displayed by WT SK-BR-3 (Figure 4B). These data suggested that ERBB1 cooperates with ERBB2 in the Tz-induced CDR formation in an ERK1/2-independent manner, and that ERBB2 is likely the main activator of the ERK1/2 signaling under these conditions. Figure 5D shows the extent of the ERBB1 expression inhibition obtained in our experimental conditions.

### Overexpression of p140Cap recapitulates the inhibitory effect of ERBB1-depletion on CDR formation and induces inactivation of cofilin

**Figure 6:**
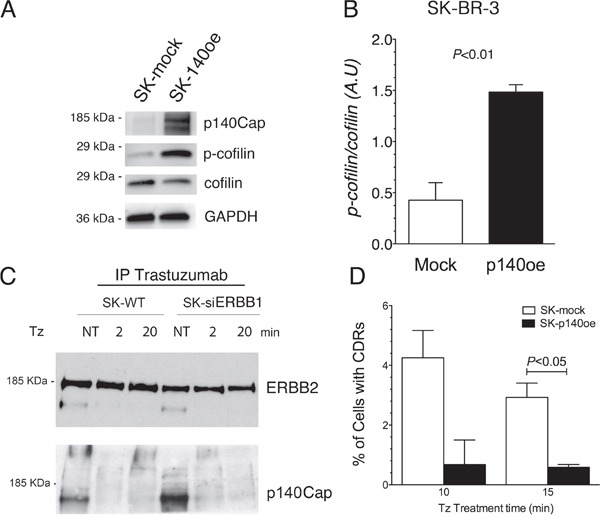
p140Cap co-immunoprecipitates with ERBB2, its overexpression recapitulates the inhibitory effect of ERBB1-depletion on CDRs formation and induces cofilin phosphorylation **(A)** SK-p140oe and SK-mock whole cell lysates were analyzed for p140Cap expression levels, cofilin phosphorylation (p-cofilin) and for total cofilin. **(B)** Histogram showing the ratio of phosphorylated/total cofilin as determined by immunoblot analysis in SK-mock and SK-p140oe cells. Bars represent the average ± SD of 3 independent experiments. **(C)** ERBB2 in SK-BR-3 wild type (SK-WT) and SK-siERBB1 was immunoprecipitated with Tz and ERBB2 and p140Cap were detected by immunoblot analysis. **(D)** Transduced SK-BR-3 cells overexpressing p140 (SK-p140oe) and SK-mock transduced cells were treated with Tz for various time points as indicated (in min) and the histogram showing the percentage of cells with CDRs at each time point. Histogram bars represent the average ± SD of the percentage of cells with CDRs, observed in 2 replicates. At each time point at least 500 cells were analyzed.

It has been previously shown that ERBB1 activity is antagonized by p140Cap protein [36], an adaptor protein involved in actin remodeling [32], which co-immunoprecipitates with ERBB1 [36]. In particular, p140Cap was described as an oncosuppressor acting as a negative regulator of cell motility and invasion [37], and ERBB2-mediated breast cancer progression [38]. To test the involvement of p140Cap in CDR formation induced by Tz, and its relationship with ERBB1 and ERBB2 signaling, we first evaluated the expression of p140Cap in WT SK-BR-3 and in SK-siERBB1 by immunoblot analysis of whole cell lysates. This analysis revealed that p140Cap was expressed at a lower level in WT SK-BR-3, compared to SK-siERBB1 (Supplementary Figure 6). Subsequently, we explored whether the p140Cap negative effect on CDR formation was due to its ability to induce cofilin phosphorylation as previously reported [39]. Indeed, by measuring the p-cofilin/cofilin ratio in p140Cap overexpressing SK-BR-3 cells (p140oe) and mock-transduced cells by immunoblot analysis, we found that p140Cap enhanced cofilin phosphorylation thus inhibiting its activity (P<0.01) (Figure 6A, 6B).

To understand whether p140Cap interacted with ERBB2 in both SK-BR-3 WT and SK-siERBB1 cells, we performed co-IP analysis of the Tz-bound ERBB2 and found an increased p140Cap-ERBB2 co-IP in SK-siERBB1, compared to SK-BR-3 WT cells (Figure 6C).

**Figure 7:**
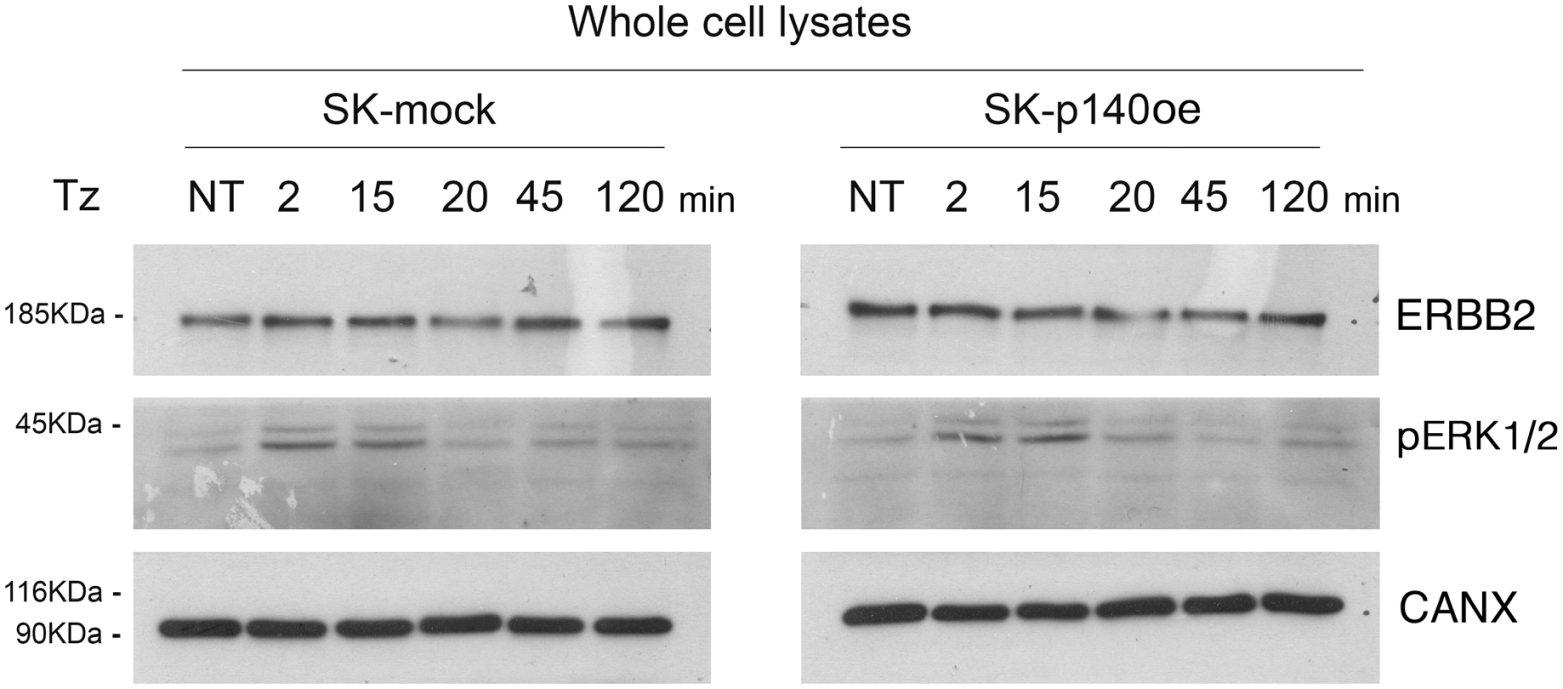
p140Cap does not induce ERK1/2 phosphorylation Immunoblot analysis of ErbB2 expression and (pERK1/2) in SK-p140oe and SK-mock cell lysates. Calnexin (CANX) was used as loading control.

To evaluate the effect of p140Cap in CDR formation, we generated SK-BR-3 cell lines overexpressing the protein and the relative controls, SK-p140Cap overexpressing (SK-p140oe) and SK-mock transduced cells, respectively, and quantified the CDRs observed upon Tz treatment. These experiments showed a statistically significant reduction of the number of CDRs in SK-p140oe compared to SK-mock cells (P<0.05) after 15 min of treatment (Figure 6D). To establish whether the inhibitory effect of p140Cap on CDR formation was due to modulation of ERK1/2 signaling, we investigated by immunoblot analysis the levels of pERK1/2 and found no major differences between p140Cap-oe and mock transduced cells (Figure 7). In view of these results, we suggest that p140Cap may exert its negative activity on CDR formation independently from the ERK1/2 signaling pathway and possibly through cofilin phosphorylation.

### ERBB1 promotes Tz-induced CDR formation by inhibiting cofilin deactivation

**Figure 8:**
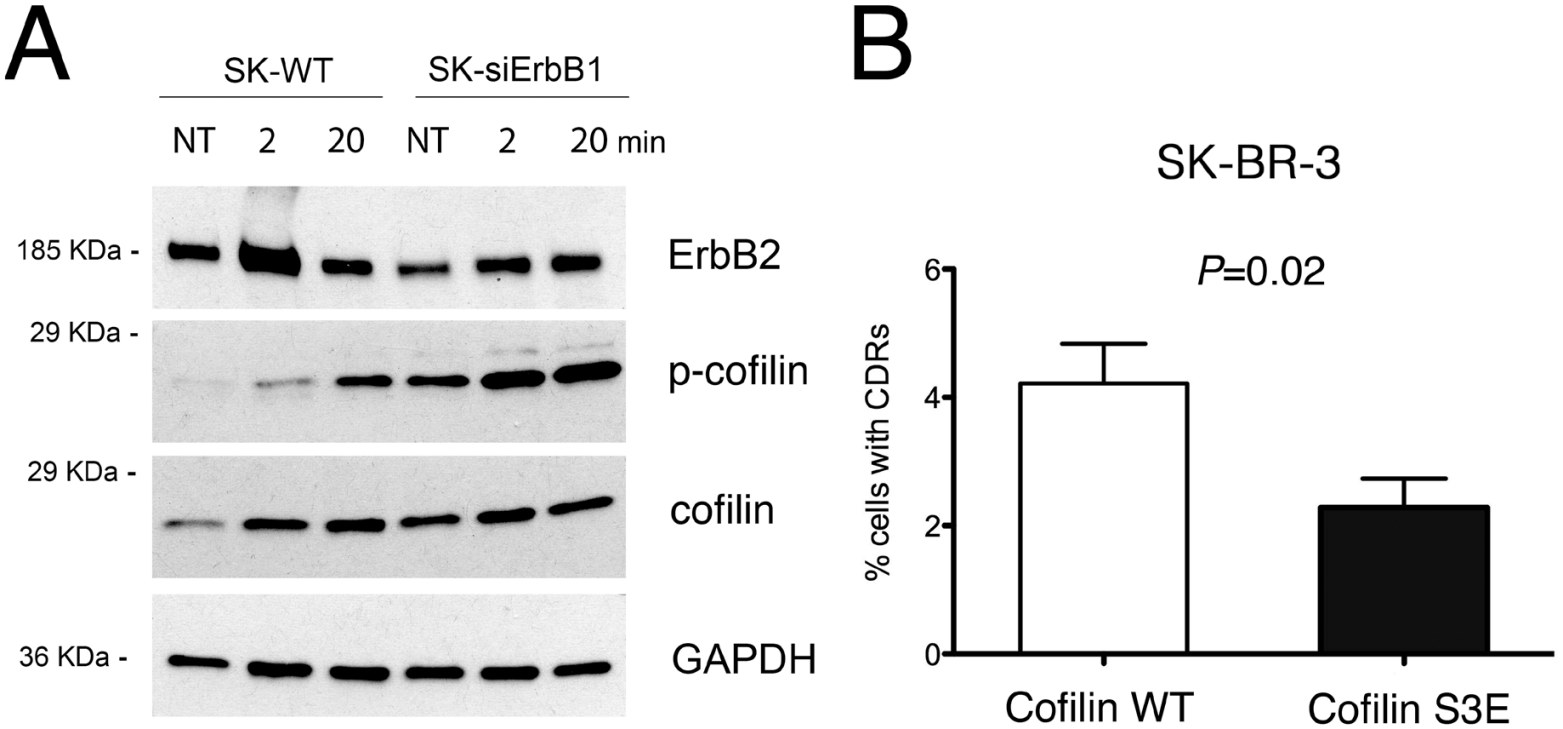
ERBB1 depletion induces cofilin-1 phosphorylation, which inhibits CDRs formation **(A)** SK-BR-3 wild type (SK-WT) or SK-BR-3 cells transfected with siRNA specific for ERBB1 (SK-siERBB1) were untreated (NT) or treated with Tz for various time points as indicated (in min) and harvested for IP and western blot analysis. Whole cell lysates were analyzed for cofilin phosphorylation (p-cofilin) and total cofilin levels (cofilin). GAPDH was used as loading control. **(B)** SK-BR-3 cells were transfected with a construct expressing wt cofilin or the S3E cofilin mutant. Histogram bars represent the average ± SD of the percentage of cells with CDRs, observed in 3 replicates. For each experiment at least 3000 cells were analyzed.

As p140Cap overexpression inhibits CDR formation and cofilin activity by inducing its phosphorylation, we analyzed the phosphorylation status of cofilin in both SK-siERBB1 and SK-BR-3 control cells transfected with a scrambled RNA oligonucleotide. The level of phosphorylated cofilin (p-cofilin) increases progressively during Tz treatment, in both silenced and control cells. However, p-cofilin reached significantly higher levels in SK-siERBB1, compared to SK-BR-3 cells (Figure 8), thus suggesting that ERBB1 signaling promoted cofilin activity by inhibiting its phosphorylation. Overall, these data suggest that upon Tz treatment of SK-BR-3 cells, ERBB1 negatively controls cofilin phosphorylation, thus promoting the actin filament severing and remodeling necessary to control CDR formation.

To test whether cofilin phosphorylation is sufficient to impair CDR formation, we expressed a phosphomimetic S3E mutant of cofilin SK-BR-3 cells, and scored the number of CDRs induced by Tz treatment, compared to cells transfected with WT cofilin, in three pooled independent experiments. The data showed a significant (P=0.02) reduction of CDR formation in SK-BR-3 cells expressing the phosphomimetic S3E mutant (Figure 8B), compared to control cells. The expression of both cofilin WT and of the phosphomimetic S3E mutant was assessed by immunofluorescence analysis (Supplementary Figure 7).

Taken together, our results show that Tz-induced formation of transient CDRs depends on the activation of ERK1/2 signaling triggered by ERBB2 and on ERBB1-mediated maintenance of cofilin activity.

## DISCUSSION

Trastuzumab is a humanized antibody directed against ERBB2 that revolutionized the treatment of ERBB2 breast cancers [10]. The introduction of Tz therapy and, more recently, of the first successful ERBB2-targeted antibody–drug conjugate (ADC), trastuzumab emtansine (T-DM1, Kadcyla, Genentech) markedly improved the poor prognosis associated with ERBB2-amplified breast cancers [40] [41] [42]. Notwithstanding, as the very early signaling events generated by Tz in targeted cells are poorly studied, we performed biochemical and morphological analyses in the ERBB2 overexpressing breast cancer cell line SK-BR-3 to determine the ERBB2 and ERBB1 signaling contribution in the context of an ERBB2/ERBB1 heterodimer. In particular, we observed that Tz induces CDR formation.

The formation of CDRs can be elicited by growth factors targeting RTKs [19]. Under these conditions, CDRs are proposed to act as spatially restricted signaling microdomains and as endocytic platform for receptor degradation [43]. Although CDRs significantly contribute to EGF-mediated ERBB1 internalization [37], here we observed poor ERBB2 internalization upon Tz-treatment as reported by others [17] [44]. These data support the hypothesis that Tz-induced CDRs may represent a transient but specific ERBB2/1 signaling platform in *in vitro* models. As little is known about the mechanism of their formation, it is important to determine the signaling pathways that are involved in Tz-induced CDR formation. N-WASP function, by binding to the RTK adaptor protein GRB2 [45], upstream of RAS-MEK mediated activation of ERK1/2, is critical for robust dorsal ruffles formation.

Here, we show for the first time that heterodimers formed by ERBB2/ERBB1 generates distinct signals, each necessary but not sufficient to induce the PM and cytoskeletal remodeling required for CDR formation. Firstly, we show that inhibition of the Tz dependent ERK1/2 phosphorylation via the MEK inhibitor U0126 impairs the N-WASP-ERBB2 interaction and the formation of CDRs. Because ERBB1 silencing is ineffective on ERK1/2 phosphorylation, we reason that this pathway only depends on the ERBB2 kinase activity. Secondly, we demonstrate that ERBB1 silencing completely abolished CDR formation, suggesting that a different ERBB1 signaling module is required for this process.

As actin-remodeling and the severing of pre-existing actin filaments are necessary for CDR formation, we concentrated our attention on a key protein that promotes these events, i.e cofilin [46]. The actin-severing activity of cofilin is inhibited by phosphorylation [47]. Indeed, we show that ERBB1 silencing increased the levels of p-cofilin, suggesting that the ERBB1 signaling module leading to CDR formation included maintenance of cofilin in a dephosphorylated and active status. The role of the cofilin/p-cofilin ratio in activating/inhibiting CDR formation upon Tz treatment was confirmed by the inhibition of CDR formation observed in cells overexpressing a cofilin phosphomimetic mutant.

We also suggest that the link between ERBB1 signaling and cofilin activity may be the p140Cap oncosuppressor protein. Indeed, SK-BR-3 cells overexpressing p140Cap display a lower percentage of cells with CDRs and an increased level of p-cofilin in the absence of changes of ERK1/2 phosphorylation levels compared to controls. Therefore, it appears that p140Cap has an inhibitory role on cofilin activity, and eventually on the actin severing process necessary to CDR formation. As silencing of ERBB1 increases both the levels of p-cofilin and of p140Cap interaction with ERBB2, as demonstrated by co-IP assays, we suggest that upon Tz binding, the ERBB1 function in the ERBB2/ERBB1 heterodimer is to impair the p140Cap-mediated cofilin inactivation, by displacing p140Cap from ERBB2.

In conclusion, the integration of these distinct signaling pathways is required to generate a specific crosstalk that is necessary to promote CDR formation. These data represent the first identification of an ERBB2 specific signaling activity in an ERBB heterodimer context.

## MATERIALS AND METHODS

### Cell culture and drug treatments

Breast cancer cell line SK-BR-3 and ZR751 were obtained from ATCC. SK-p140 and SK-mock transduced cell lines were developed by Dr. Paola Defilippi. Cells were cultured in DMEM, supplemented with 10% of fetal bovine serum, 1% glutamine and penicillin and streptomycin, at 37°C in a humidified atmosphere containing 5% CO_2_.

Tz (Genentech-Roche, South San Francisco, CA, USA) was dissolved with saline solution with 0.9%NaCl in a stock concentration of 20 mg/ml, donated by the pharmacy (UFA-Unità Farmaci Antiblastici) of the IRCCS AOU - San Martino - IST. Where indicated, Tz was used conjugated with Alexa-488 using the Alexa Fluor 488 Protein Labeling Kit following the manufacturer's instructions (Molecular Probes, Eugene, OR, USA).

In all the experiments, Tz was used in a concentration of 10 μg/ml [30]. Cells were treated with Tz in DMEM serum-free at 37°C. When indicated cells were treated with 100 ng/ml EGF or 1 mg/ml transferrin.

In ERK1/2 inhibition experiments, cells were pre-incubated with 10μM U0126 (Promega, Madison, WI, USA), in DMEM serum-free at 37°C for 20 minutes, and maintained at the above concentration during the Tz treatment.

### Immunofluorescence analysis

Cells were fixed in 3% paraformaldheyde (PFA) in phosphate-buffered saline (PBS) pH7.4 and then quenced with 30mM NH_4_Cl. After permeabilization with 0.2% saponin/PBS, Tz (10 μg/ml) or mouse monoclonal anti-ERBB2 9G6 (sc-08, Santa Cruz, Santa Cruz, CA, USA) were used as primary antibody in cells followed by detection with an anti-human Cy2 and anti-mouse Cy2 or Cy3 antibodies, respectively.

The following primary antibodies were incubated for 20 min in 0.2% saponin/PBS: rabbit anti-cortactin (ab11066, Abcam, Cambridge, UK), mouse anti-ERBB1 (Ab 108, kindly provided by IFOM). The secondary antibodies were incubated for 20 min in 0.2% saponin/PBS: Cy3-conjugated donkey anti-mouse IgG (Jackons ImmunoResearch Laboratories, West Grove, PA, USA) and Alexa488-cojugated goat anti-human IgG (Molecular Probes). Where indicated, cells were treated with Tz conjugated with Alexa488 or Alexa555.

The F-actin staining was detected directly with 7μM Phalloidin-TRITC (P1951, Sigma-Aldrich, St. Louis, MO, USA) after cells fixation and permeabilization.

For nuclear staining, DAPI (Sigma) at 100 ng/ml was used for 5 min prior to mounting. The coverslips were mounted using Mowiol 4-88 reagent (Calbiochem, San Diego, CA, USA). Image acquisition was perfomed with an Olympus IX70 epifluorescence microscope. Images were captured under oil with a 63x plan apochromat objective. When indicated, images were acquired at 37°C with TCS-SP2 AOBS confocal microscope station (Leica Microsystems, Wetzlar, Germany).

### Immunoprecipitation (IP) and western blot analysis

Protein cell extracts and SDS Polyacrylamide gel electrophoresis were performed using standard protocols [31]. Protein quantification was performed using Bradford protein assay (BioRad, Hercules, CA, USA). Tz administered to live starved cells (at 10μg/ml of final concentration) for 2 min or 20 min (serum free medium) was used as a primary anti-ERBB2 antibody. To immunoprecipitate ERBB2 from control Tz-untreated and starved cells we first performed cell lysis and subsequently added Tz to the cell lysate (at 10μg/ml of final concentration). Protein A-Sepharose CL-4B (GE Healthcare, Piscataway, NJ, USA) was added to the cell lysate to immunoprecipitate Tz bound to ERBB2. For ERBB1 IP, we used agarose-conjugated anti-EGFR (sc-03 AC, Santa Cruz Biotechnology, Santa Cruz, CA, USA).

Samples were separated by SDS–PAGE and analyzed by immunoblotting, using as primary antibodies: rabbit anti-ERBB2 (c-18, sc-284, Santa Cruz), rabbit anti p-ERBB2 Y1248 (#2247; Cell Signaling Technology, Danvers, MA, USA), goat anti-pERK1/2 (sc-16982, Santa Cruz), rabbit anti-calnexin (sc-11397, Santa Cruz), rabbit anti-GAPDH (#2118, Cell Signaling), mouse anti-cofilin (1G6A2, Proteintech, Rosemont, IL, USA), rabbit anti-p-cofilin (#3311, Cell Signaling), rabbit anti-cortactin (#3503, Cell Signaling), mouse anti-p140Cap (generated by Dr. Paola Defilippi's laboratory) [32]. Secondary antibodies were horseradish peroxidase-conjugated: anti-mouse (Molecular Probes, Thermo Fisher Scientific, Waltham, MA, USA), anti-rabbit (Molecular Probes) and anti-goat (Santa Cruz) and the detection of proteins were performed with ECL Detection Reagent (GE Healthcare).

Densitometric analysis of western blots was performed with ImageJ Gel Analysis software.

### RNA interference

SK-BR-3 cells were transiently transfected with two siRNA Stealth oligos (Invitrogen, Carlsbad, CA, USA) anti-ERBB1:#1 (5′-CCGCAGCAUGUCAAGAUCACAGAUU-3′), and #2 (5′-CCACCGUGGCUUGCAUUGAUAGAAA-3′) using Lipofectamine 2000 accordingly to the manufacturer's instructions (Invitrogen). All the experiments were performed using cells transfected with a scrambled (SCR) oligo as control (5′-CCGACGUGUAACUAGCACGACAAUU-3′). Briefly, cells were subjected to two rounds of transfection, 24 and 48 hours after plating, and were treated and processed 72 hours after the first round of transfection.

### Transfection of expression vectors

Construct transfections were performed using Lipofectamine 2000, accordingly to the manufecturer's instructions (Invitrogen). Constructs employed in this study were: a N-WASP:GFP chimera [33] and the pmRFP-N1 human cofilin WT and S3E, which were acquired from Addgene (plasmid # 50856 and # 50858, Cambridge, MA, USA).

Localization of N-WASP:GFP in epifluorescent microscopy was performed 48h after cell transfection while CDRs were analyzed in cofilin WT:RFP and cofilin S3E:RFP chimera expressing cells 18h after transfection.

### ERBB2 plasma membrane redistribution and Circular Dorsal ruffling assay and statistical analysis

To analyze the number of cells showing a single CDR two observers evaluated slides independently and at least 500 cells, for each experimental condition, were analyzed. All parameters measured are presented as mean ± standard deviation and were analyzed with the Student's t-test using a two-tailed distribution. P values < 0.05 were considered statistically significant.

## SUPPLEMENTARY MATERIALS FIGURES



## Abbreviations

Tz: Trastuzumab
RTKs: receptor tyrosine kinases
PM: plasma membrane
CDR: circular dorsal ruffle
IP: immunoprecipitation
GFP: green fluorescent protein.
