# Supplementary material for: Cooperative but distinct early co-signaling events originate from ERBB2 and ERBB1 receptors upon trastuzumab treatment in breast cancer cells
Source: Oncotarget. 2017 May 8;8(36):60109–22. doi: 10.18632/oncotarget.17686 (PMC5601125; doi:10.18632/oncotarget.17686)
Supplement: Supplementary file 1 [file oncotarget-08-60109-s001.pdf]

Cooperative but distinct early co-signaling events originate from ERBB2 and ERBB1 receptors upon trastuzumab treatment in breast cancer cells

SUPPLEMENTARY FIGURES

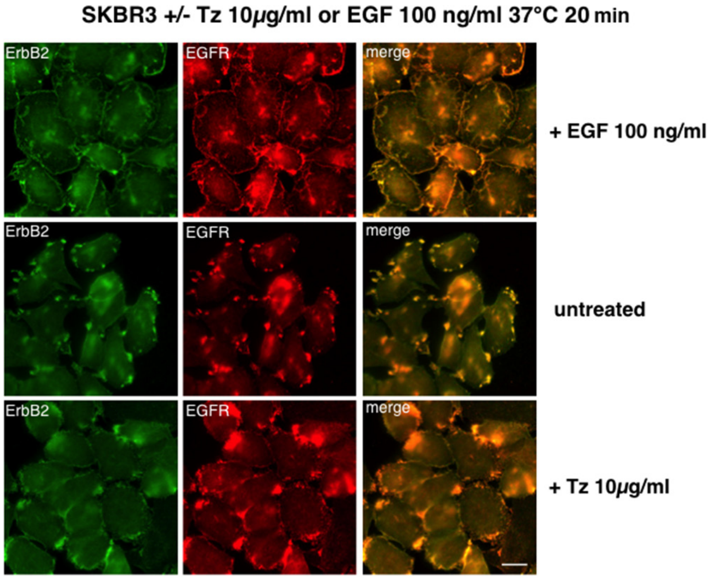

Supplementary Figure 1: ERBB1 and ERBB2 codistributes in SK-BR-3 cells after CDRs disappear yielding a diffuse PM staining. SK-BR-3 cells were untreated or treated with 100 ng/ml EGF or with 10 µg/ml Trastuzumab (Tz) for 20 min, fixed and processed for immunofluorescent detection of ErbB1 (EGFR) (red signal) and ErbB2 (green signal). Scale bars = 10 µm.

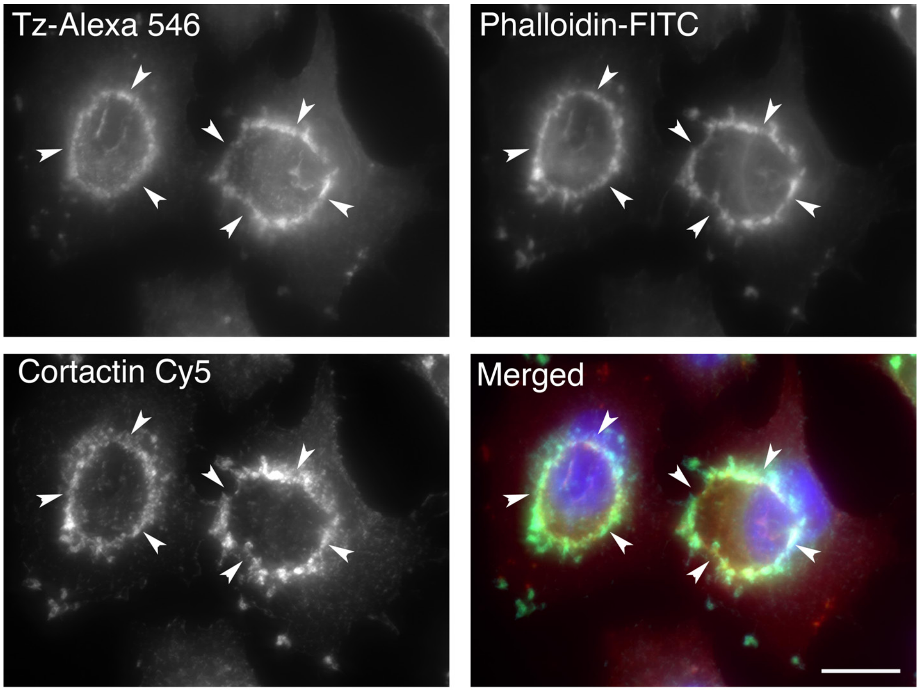

Supplementary Figure 2: Cortactin colocalizes with F-actin and ERBB2 in CDRs on the plasmamembrane of SKBR3 treated for 10 min with Tz. Immunofluorescence analysis of ERBB2, F-actin and cortactin localization. ERBB2 was revealed with Tz alexa 546, FITC-phalloidin was used for F-actin, and a Cy5 conjugated secondary antibody was used for cortactin. Arrow heads point to CDRs. Scale bars = 10 µm.

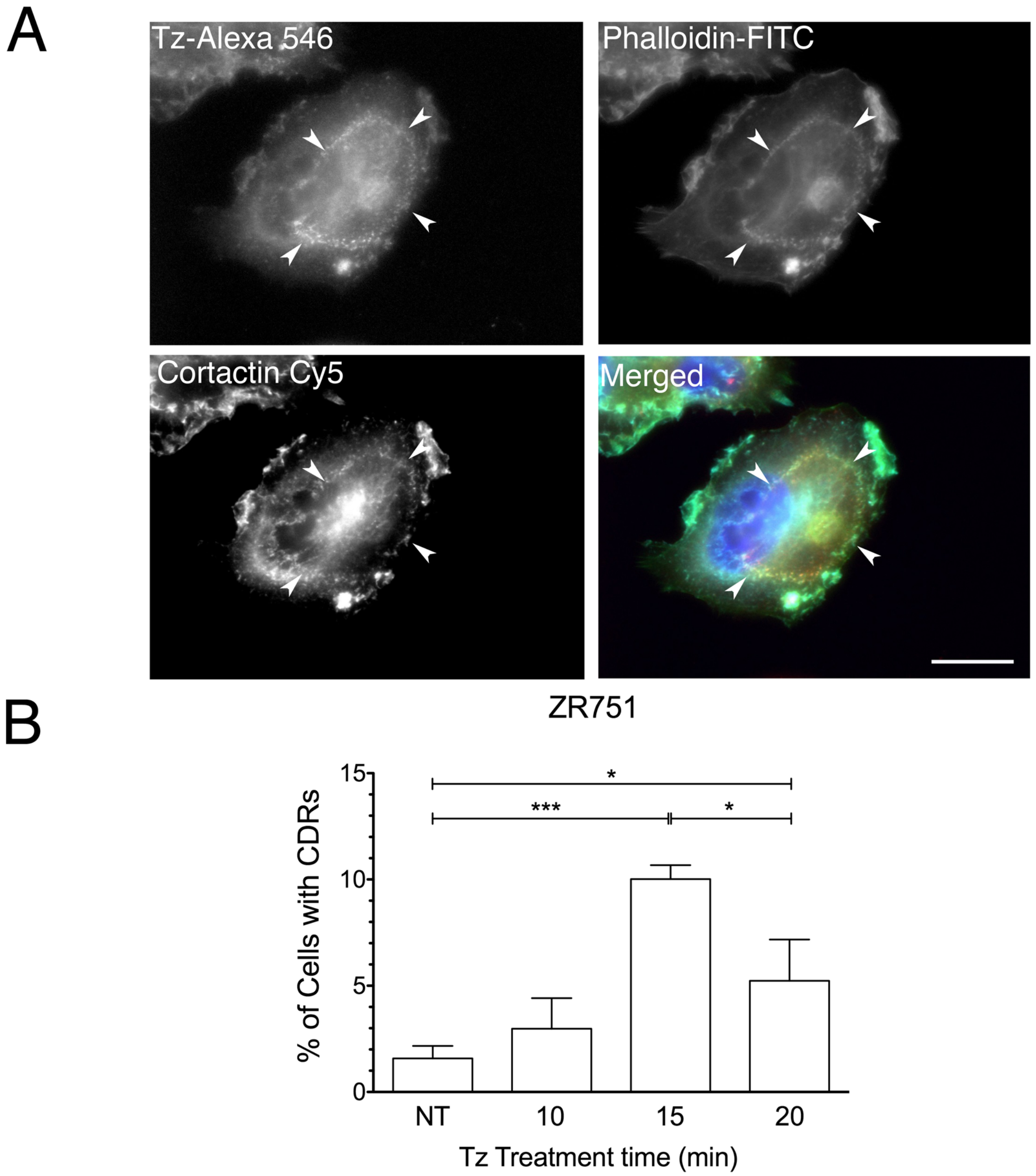

Supplementary Figure 3: Breast cancer cell line ZR7851 shows CDR formation after treatment with Tz. **(A)** Immunofluorescence analysis of ZR7851 treated for 10 min with Tz. ERBB2 was revealed with Tz alexa 546, FITC-phalloidin was used for F-actin, and a Cy5 conjugated secondary antibody was used for cortactin. Arrowheads point to a CDR. Scale bars = 10 µm. **(B)** Histogram of the percentage of ZR7851 cells showing CDRs at each time point. Bars represent the average ± SD of the percentage of cells with CDRs, observed at each time point pooled from 3 independent experiments. At each time point at least 500 cells were analyzed. * $P<0.05$; *** $P<0.0001$.

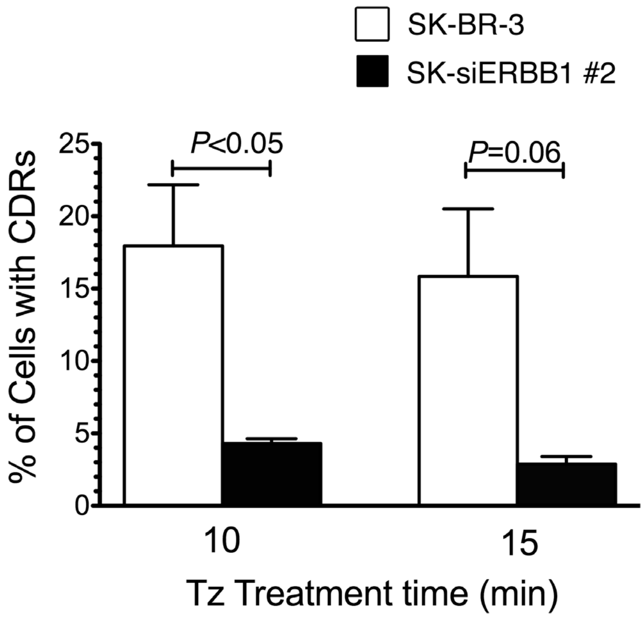

Supplementary Figure 4: SK-BR-3 wt or SK-BR-3 cells transfected with the siRNA #2 (see Material and Methods) specific for ERBB1 (SK-siERBB1) were treated with Tz for various times as indicated (in min). Histogram of the percentage of cells showing CDRs at each time point. Bars represent the average ± SD of the percentage of cells with CDRs, observed at each time point pooled from 3 independent experiments. At each time point at least 500 cells were analyzed.

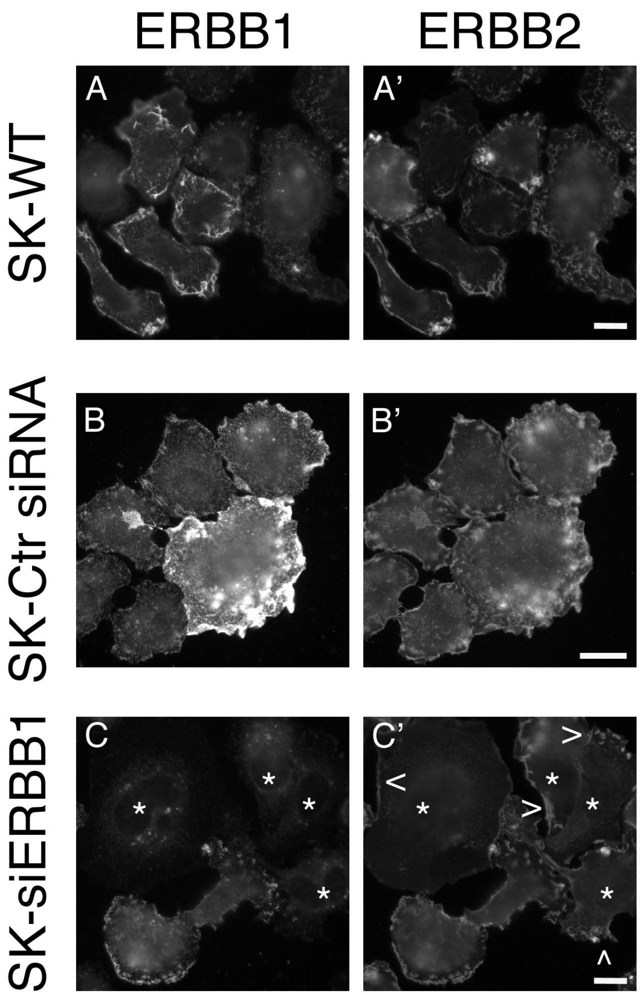

Supplementary Figure 5: ERBB2 is present on the plasmamembrane of SK-BR-3 cells in which the expression of ERBB1 is inhibited by RNA interference. SK-BR-3 WT cells, SK-BR-3 cells transfected with a control scrambled RNA oligonucleotide (see Materials and Methods) or with a ERBB1 specific siRNA #1 (SK-siERBB1) (see Materials and Methods) were treated with Tz for 2 min and evaluated by immunofluorescence analysis using a FITC-conjugated ERBB1 **(A-C)** antibody and a TRITC-conjugated ERBB2 **(A'-C')**. Cells with no o low ERBB1 expression in plasmamembrane are indicated by asterisks (*). Arrowheads point to ERBB2 localized on the plasmamembrane. Scale bars = 10 µm.

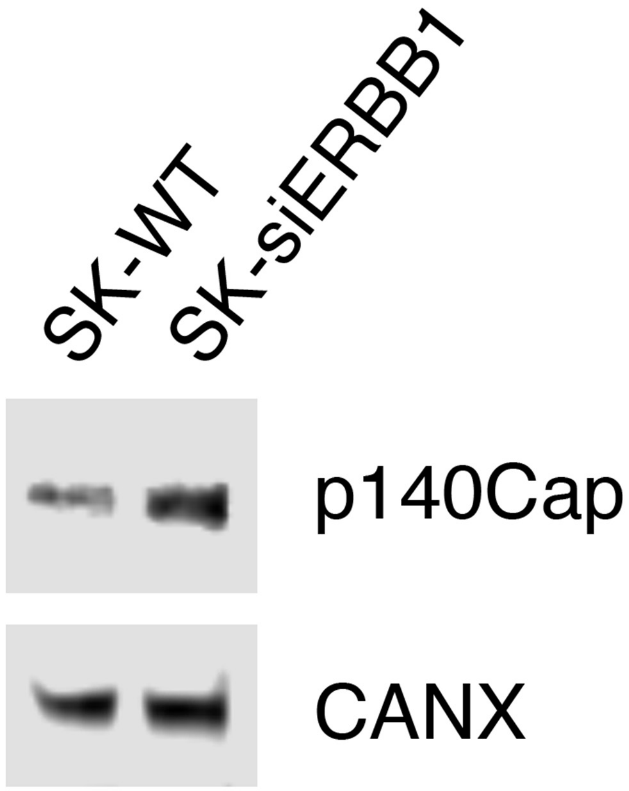

Supplementary Figure 6: Immunoblot analysis of p140Cap expression in SK-BR-3 WT cells (SK-WT) and in SK-BR-3 cells in which the expression of ERBB1 was inhibited by RNA interference (SK-siERBB1) with siRNA #1 (see Materials and Methods). Calnexin (CANX) was used as loading control.

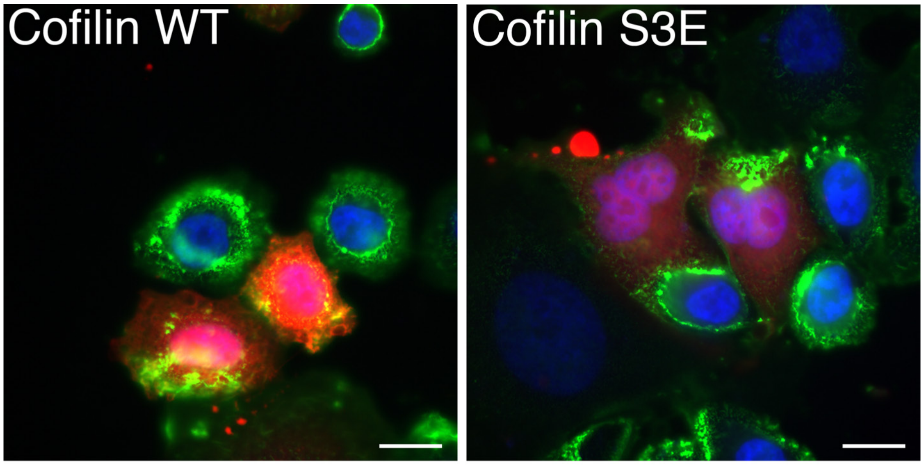

Supplementary Figure 7: Epifluorescence microscopy analysis shows expression of cofilin WT:RFP and cofilin S3E:RFP chimera (red signal) in SK-BR-3 cells. F-actin was stained with FITC-conjugated Phalloidin (green signal). Nuclei were stained with DAPI (blue signal). Scale bars = 10 µm.
